# Sensitivity of speleothem records in the Indian Summer Monsoon region to dry season infiltration

**DOI:** 10.1038/s41598-019-41630-2

**Published:** 2019-03-25

**Authors:** Elli R. Ronay, Sebastian F. M. Breitenbach, Jessica L. Oster

**Affiliations:** 1 Vanderbilt University, Department of Earth and Environmental Sciences, Nashville, TN 37212 USA; 2 Ruhr-University Bochum, Institute for Geology, Mineralogy & Geophysics, Bochum, Germany

## Abstract

In climates with strongly seasonal rainfall, speleothem-based paleoclimate reconstructions are often thought to reflect wet season conditions, assuming a bias toward the season with greater water supply. This is particularly true in monsoon regions, where speleothem records are interpreted to document monsoon strength changes on multiple timescales. Dry season infiltration variability and rainfall seasonality are not typically considered in these reconstructions, even though cave ventilation could bias speleothem growth toward the cooler season. To investigate the influence of dry season infiltration on speleothem geochemistry, we combine a modern, sub-seasonally resolved trace element record from Mawmluh Cave in Northeast India with forward modeling experiments. We find that variations in the amplitude of seasonal signals in speleothem Mg/Ca, which reflects prior carbonate precipitation, are more sensitive to dry season rather than monsoon season infiltration. This sensitivity may be enhanced by dry season cave ventilation. The Mawmluh speleothem Mg/Ca record is consistent with increased dry season rainfall during the 1976–1998 warm phase of the Pacific Decadal Oscillation relative to 1964–2013. Our work demonstrates the importance of considering non-monsoon season rainfall when interpreting speleothem paleoclimate records and suggests that trace elements could provide insight into periods of enhanced dry season infiltration in monsoonal climates.

## Introduction

The Indian Summer Monsoon (ISM) is a critical component of the climate system in South Asia, delivering 70–80% of annual rainfall between June and September (JJAS). The ISM drives the South Asian agricultural economy and its strength has significant societal impacts resulting from both water scarcity and disastrous excess^1,2^. The amount of monsoonal rainfall is modulated by the El Niño-Southern Oscillation (ENSO), including the flavor of El Niño events, and decadal scale variability in the Pacific^3–7^. Weak ISMs have historically been associated with Central Pacific (CP) El Niño events^8^ and this is enhanced during the warm phase of the Pacific Decadal Oscillation (PDO)^9^.

Acute water shortages occur across India during the dry season following years of weak ISM^10^. These shortages are exacerbated in years of below-average dry season (winter) rainfall. In Northeast (NE) India, winter atmospheric circulation is governed by the NE winter monsoon, which brings cold, dry air from the Tibetan plateau. Winter rainfall can arise from storms trending from Central Asia called western disturbances^11,12^. Although it has been suggested that PDO variations can modulate these western disturbances^11,13^, comparatively little is understood about winter rainfall and its relationship to broader internal ocean-atmosphere variability in NE India. A thorough comprehension of the controls on dry season rainfall in NE India in addition to ISM strength will have important implications for water use planning and mitigative actions in light of current climate change.

The concern for water shortages in NE India due to climate change is reinforced by the local paleoclimate record. Stable isotope records from speleothems have been interpreted to reflect intense droughts in NE India during the Holocene that are associated with anomalous climate throughout Asia and worldwide^14^. The identification of a megadrought in NE India and other locations at 4.2 ka contributed to the recent and controversial subdivision of the Holocene and the establishment of the Meghalayan Age^15,16^. The Global Boundary Stratotype Section and Point for the beginning of the Meghalayan at 4.2 ka is defined by a shift in the δ^18^O of a speleothem from Mawmluh Cave in NE India^17,18^, interpreted to reflect the start of a prolonged decrease in ISM strength. However, isotope-enabled climate modeling suggests that speleothem δ^18^O in the broader Asian monsoon region primarily records changes in large scale atmospheric processes^19–21^, and does not provide unequivocal information about rainfall amount. Furthermore, recent work locally in NE India has demonstrated that δ^18^O in rainwater, dripwater, and speleothems from this region reflects variations in moisture transport on seasonal to millennial timescales^6,22–24^.

Additional, independent proxies are required to investigate and relate rainfall amount changes to moisture transport history. Further, it is not known how variations in rainfall seasonality are recorded in speleothems from this region or what might drive seasonality on decadal or longer timescales^24^. Pinpointing the cause(s) of changes in dry season rainfall and how this might be reflected in dripwater chemistry could significantly improve our understanding of proxy seasonality in speleothem records from monsoonal settings. Here we use a high temporal resolution (sub-annual) record of trace element ratios in a modern speleothem from Mawmluh Cave to investigate their fidelity as proxies for rainfall amount and seasonality in NE India. We further examine the relationships between Pacific climate variability and year-round rainfall in this water-sensitive region.

## Site and Sample Description

**Figure 1:**
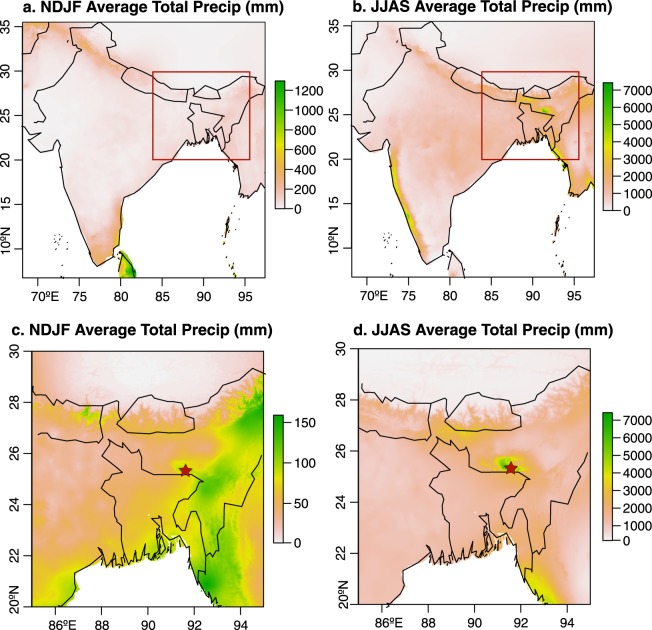
Average winter (**a,c**) and monsoon season (**b,d**) precipitation for India (**a,b**), and the area surrounding Mawmluh Cave (**c,d**). Precipitation data is the WorldClim version 2, 2.5 minute resolution climate data which consists of monthly precipitation averages computed for 1970–2000^55^. Note that each panel has an independent scale bar to preserve spatial variation in precipitation amounts. Location of Mawmluh Cave is shown by the red star. Map was created using the raster package in R^56^.

Mawmluh Cave is located on the southern margin of the Meghalaya Plateau (25°15′36″N 91°52′48″E, Fig. 1), 13.7 km from Mawsynram and 2.3 km from Sohra (Cherrapunji), two villages that alternate for the title of the wettest place on Earth^25^. The region receives 70–80% of its annual rainfall during the ISM season (JJAS) (Fig. 1), averaging 8342 mm (max. 19519 mm, min. 4493 mm) of ISM season rainfall from 1901–2014^26^. The Meghalaya Plateau is the first orographic feature that north-trending, moisture-laden ISM winds encounter as they move inland (Fig. 1), inducing orographic rainfall on its southern edge^24^. Mawmluh Cave is located 1320 m above sea level with 30–100 m of karstified limestone, dolostone, and sandstone overlying the cave, topped with 5–15 cm of soil^23,24^.

Mawmluh Cave is inaccessible during the height of the monsoon season due to flooding within the cave, precluding measurements of cave air and drip water chemistry during JJAS. However, monitoring from 2007–2014 shows cave air *p*CO_2_ is lowest during the winter months due to air temperature driven ventilation and rises through May as the monsoon season is beginning^23^. Soil *p*CO_2_ also increases slightly through the winter into May, but the increase is smaller than what is observed in cave air^23^. This *p*CO_2_ variability likely drives seasonal differences in speleothem growth rate due to differential CO_2_ degassing, such that more speleothem growth occurs during the winter dry season^27^. Dripwater monitoring indicates that the transmission of δ^18^O signals from rainwater to dripwater occurs within one month, which is evidence that seasonal-scale changes in water chemistry can be recorded in Mawmluh Cave speleothems^23^.

Stalagmite MAW-0201 was collected from the Hanging Gardens passage of Mawmluh Cave in February 2013 and was actively growing at the time of collection (Fig. S1). MAW-0201 is 22 mm long and composed of laminated aragonite, which precipitated on the base of a broken calcite stalagmite. Each ~380 μm thick lamina consists of a light-dark couplet resulting from seasonal density contrast. MAW-0201 has high uranium content (~40 ppm), which allows dating at high precision, giving errors of 1.1 to 2.7 years for the record of growth from 1964–2013^6^. Previous analysis of MAW-0201 revealed seasonal variations in speleothem δ^18^O in line with seasonal dripwater values^6^. The lighter colored, microporous laminae attributed to dry season growth have generally higher δ^18^O than the darker, denser laminae of the wet season. In four measurements of MAW-0201 laminae by two independent observers, the lighter colored laminae attributed to the dry season accounted for 61–65% of the annual growth.

## Results

**Figure 2:**
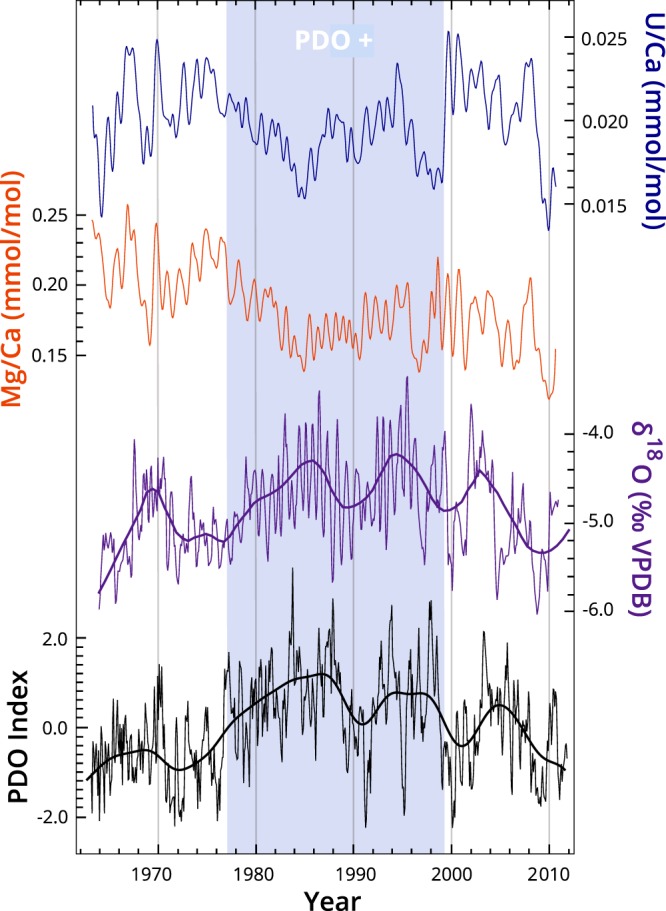
Top to bottom: Gaussian kernel smoothed U/Ca (blue) and Mg/Ca (orange), δ^18^O (purple) and PDO index (black, http://research.jisao.washington.edu/pdo/) with bold LOESS smoothed lines. The blue bar indicates the period of positive PDO index.

**Figure 3:**
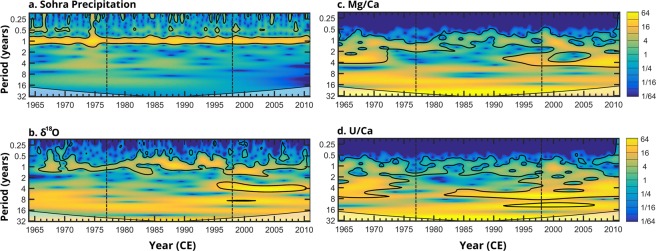
CWTs of (**a**). Sohra rainfall (http://climexp.knmi.nl), (**b**) δ^18^O, (**c**) Mg/Ca, and (**d**) U/Ca in MAW-0201. Signals outlined in black are significant at the 95% confidence interval, more yellow colors signify a stronger periodicity. The black dotted box represents the period of positive PDO index and coincides with significant changes in signal strength.

Trace element to calcium ratios Mg/Ca, U/Ca (Fig. 2), Sr/Ca, and Ba/Ca (Fig. S2) obtained with laser ablation (see methods section) show seasonal variability throughout the record and are all positively correlated with the exception of Sr/Ca and U/Ca (Table S1). Mg/Ca and U/Ca also display periods of apparent, but not significant anticorrelation for the periods of 1976–1982 and 2002–2005. Continuous wavelet transforms (CWTs) of Mg/Ca and U/Ca time series show a significant seasonal (1 year) signal consistently throughout the record (Fig. 3). The strength of this seasonal signal diminishes but remains significant between ~1976 and 1998, coincident with a reduction of the seasonal amplitudes in Mg/Ca and U/Ca and the interval of positive PDO (1977–1998)^28^. Significant ENSO-scale (2–8 year) periodicities are evident prior to 1972 in Mg/Ca and 1976 in U/Ca. After ~1981, 4–8 year periodicities return in the U/Ca time series. After 1990, 2–4 year periodicities become strong and significant in the Mg/Ca record. Similar patterns are seen in the δ^18^O CWT, where ENSO-scale periodicities are insignificant when the PDO index is positive^6^.

## Discussion

The positive correlations between Mg/Ca, Sr/Ca, and Ba/Ca suggest that seasonal variations in these element ratios likely reflect changes in the degree of prior carbonate precipitation, or PCP^29–31^. When PCP occurs, Ca is preferentially incorporated into both aragonite and calcite prior to arrival on the stalagmite apex. If calcite is precipitating, the concentrations of Mg, U, Sr, and Ba increase in the fluid relative to Ca because of their <1 distribution coefficients (*D*_X_)^32^. If aragonite is precipitating, the fluid Mg/Ca will increase, but U/Ca and Sr/Ca will decrease (the behavior of Ba is still unclear)^33^. The observed positive correlation of U/Ca with Mg/Ca and Ba/Ca in MAW-0201 throughout most of the record suggests that U is primarily sourced from the bedrock rather than from the soil, as a soil U source would also result in an anti-correlation between U/Ca and the other trace elements. With a soil source, peaks in U/Ca would occur during the ISM season, when components from the soil are more likely to be flushed into the cave, while PCP is thought to be minimal^31^. The positive correlation between U/Ca and the other trace element ratios also suggests that U/Ca is sensitive to PCP and that calcite is the primary form of PCP in the Mawmluh Cave epikarst system. The exceptions to this are the periods when Mg/Ca and U/Ca display negative correlations (1976–1982 and 2002–2005) which could reflect intervals of prior aragonite precipitation (PAP) in the epikarst. During the 1976–1982 period of apparent anticorrelation between U/Ca and Mg/Ca, Ba/Ca is also anticorrelated with U/Ca. While the distribution coefficient for Ba in aragonite is not entirely constrained, its behavior in Mawmluh Cave speleothems suggests *D*_Ba_ < 1, and its anticorrelation with U/Ca supports the idea that, periodically, aragonite precipitates in the epikarst^33^.

The extent of PCP is primarily controlled by water residence time in the epikarst, which is largely a function of effective rainfall, cave ventilation, and precursory epikarst saturation^30,34,35^. Cave monitoring studies have shown that seasonal cave ventilation modulates CO_2_ degassing in the epikarst and cave ceiling in ways that may be unrelated to infiltration rates^35^. However, in caves where seasonal differences in ventilation are small, rainfall amounts can drive trace element variability^31^. Available measurements of soil and cave *p*CO_2_ from Mawmluh suggest that soil *p*CO_2_ variability is likely small (441–481 ppm, with one outlier measurement of 5936 ppm)^23^. Cave air *p*CO_2_ near the collection site of MAW-0201 also shows minimal variability in the winter and early spring months (~370 to 530 ppm). Although direct measurements of monsoon season *p*CO_2_ have not been possible, both soil and cave air *p*CO_2_ increase in May with the onset of the monsoon season, though the increase in cave air *p*CO_2_ is substantially larger than the soil *p*CO_2_ increase. This high cave *p*CO_2_ would suppress PCP as well as speleothem growth at the collection site during the monsoon season. Thus, the seasonal signal in trace element to Ca ratio variability likely reflects a combination of infiltration and ventilation forcing that leads to enhanced PCP (and higher element ratios) during the dry winter months and decreased PCP (and lower element ratios) during the monsoon season.

In addition to the seasonal signal of variability, Sr/Ca and Ba/Ca show a decreasing trend throughout the record, which is not present in U/Ca, and only evident in Mg/Ca until ~1985. The meteorological record of annual rainfall from 1964–2013 shows no significant long-term trend (Fig. S3), which implies that the decreasing trends in element ratios are caused by a process unrelated to rainfall. For example, exposure of fresh dolomitized hostrock along infiltration flow paths due to seismic activity or land-use change may explain why the decrease in Mg/Ca ceases around 1985. A 5.1 magnitude earthquake that occurred ~30 km away from Mawmluh Cave in September 1984 may have exposed fresh dolomite along the flow path^36^. After exposure, fresh dolomite surfaces, having higher Mg concentration than long-leached hostrock along the flow path, may allow more Mg to enter the dripwater. If the Sr/Ca and Ba/Ca are not replenished by the new exposure, as with Mg from dolomite, their ratios would continue to decrease^37^.

### Evaluating the influence of seasonal infiltration on speleothem Mg/Ca

The variability in seasonal amplitudes of trace element ratios and the changes we see in the strength of seasonal periodicities occur in concert with changes in the phase of PDO and winter rainfall amounts. Between 1977 and 1998, during the warm phase of PDO, average December rainfall at Sohra tripled, rising from 10 mm/month before 1977 to 31 mm/month between 1977–1998, and then decreased to 7 mm/month after 1998 (Fig. S4). Based on measurements of cave and soil *p*CO_2_, we predict enhanced speleothem growth in winter, despite minimal rainfall and drip rates. Below, we test how these variables may affect seasonal amplitudes of trace element ratios. In particular, we evaluate how seasonal changes in rainfall may be recorded in speleothem Mg/Ca when speleothem growth is biased towards the dry season. We focus on Mg/Ca because its predicted behavior in response to PCP will be similar regardless of whether PCP in the epikarst occurs as calcite or aragonite.

To test the differential effects of changing ISM and winter rainfall on Mg/Ca in Mawmluh Cave speleothems, we use the forward model I-STAL^38^. I-STAL models the effects of water-rock interaction and PCP on dripwater chemistry. The model uses carbonate trace element distribution coefficients and measured cave variables including drip interval (time between drips), cave air *p*CO_2_, temperature, and initial CaCO_3_ saturation, or [Ca], to estimate trace element ratios in the resulting dripwater. We forward model Mg/Ca in a pseudo-stalagmite during years characterized by strong versus weak ISM rainfall and dry versus wet winters. We use an initial [Ca] value of 153 ppm, which is the median [Ca] measured in Mawmluh Cave dripwater (see Fig. S5). We also test the sensitivity of the model to initial [Ca] by using the range of values measured in Mawmluh Cave (111–420 ppm) (Fig. S5). We use the temperature dependent calcite distribution coefficient (*D*_Mg_) from Day & Henderson (2013) for all runs assuming calcite is the primary form of PCP. A Mawmluh Cave specific *D*_Mg_ for aragonite^33^ was used for runs testing the effects of PAP.

Rainfall amount is expressed in I-STAL through the drip interval parameter. To determine reasonable drip intervals for Mawmluh Cave, we use drip rate monitoring and daily Sohra rainfall data from Breitenbach *et al*.^23^ to estimate an empirical relationship between monthly-integrated rainfall and drip interval for our site. We compare the total rainfall amount for the given month to all measured drip intervals within that month, which gives us a spread of drip intervals that were associated with rainfall amount in that month (Fig. S6). This relationship was then used to estimate drip interval, *I* (s), from a given month of rainfall, *R* (mm/month), shown by

1 $$I=337.25{e}^{-0.002R}.$$

Using this relationship, we model an exceptionally strong ISM with monthly drip intervals ranging from 0.007–1.8 s, drawn from the maximum (non-outlier) rainfall amounts recorded for each month from the rainfall record in Sohra, India from 1901 to 2014 (Fig. S7). The total JJAS rainfall found by summing these monthly maxima (16351 mm) is 3.9 standard deviations higher than mean local JJAS rainfall and higher than any of the ISMs on record in Sohra except for 1974 (19519 mm). To model a weak ISM, we use the first quartile value of each month from the 1901–2014 rainfall record. These rainfall amounts correspond to 5.6–96 s drip intervals during the ISM months. Summing these monthly rainfall amounts results in 5952 mm JJAS rainfall, which is 1.2 standard deviations lower than mean JJAS rainfall. We chose the first quartile value rather than the minimum values of JJAS rainfall, as summing the minima leads to an unrealistically small amount of ISM rainfall (2347 mm), which is far less than the weakest monsoon on record (4493 mm in 1961).

At our site “dry winters” are characterized by zero NDJF rainfall. We model this with a drip interval of 337 s, which is the zero rainfall y-intercept in our calibrated Equation 1 and falls within observed drip intervals for zero rainfall months. In the model, we allow drip interval to increase to 345 s and 350 s in the third and fourth months of <1 mm rainfall. In the “wet winter” scenarios, we use the maximum (non-outlier) values of NDJF rainfall from 1901–2014, corresponding to drip intervals of 247–321 s and a total of 375 mm NDJF rainfall. For all other months (MAM and O), we use the median value of rainfall from 1901–2014.

**Figure 4:**
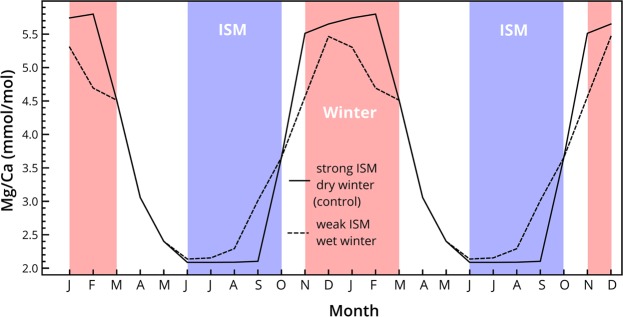
Mg/Ca from two-year I-STAL forward modeling experiments using *p*CO_2_ and drip intervals from Breitenbach *et al*.^23^. Strong ISM with dry winter control in solid line, weak ISM with wet winter in dashed line. The wet winter contribution to amplitude decrease is 16.5%, weak ISM contribution to amplitude decrease is 5.8%. I-STAL inputs listed in Table S2.

We use a hypothetical year with a strong ISM and dry winter as the control for the I-STAL forward modeling experiments. In this control run we first take the square deviation of each month from the mean of the year’s modeled dripwater Mg/Ca, then take the root of the mean of these monthly square deviations to derive an amplitude of Mg/Ca for the control scenario. We repeat this root-mean-square deviation (RMSD) process for the weak ISM and wet winter experiments, which we then compare with the control Mg/Ca values (Fig. S8). All experiments result in decreased seasonal amplitude relative to the control, meaning the weak ISM experiment increases summer PCP and raises the summer-associated Mg/Ca trough, while the wet winter decreases PCP and lowers the dry season peak (Fig. 4). The weak ISM model (5952 mm JJAS rainfall) results in a 5.8% decrease in amplitude. The wet winter experiment (375 mm NDJF rainfall) results in 16.5% RMSD decrease in amplitude. Together, the weak ISM and wet winter scenario results in a 22.3% amplitude decrease compared to the strong ISM and dry winter control (Fig. 4). When using the minimum (111 ppm) and maximum (420 ppm) measured dripwater Ca as the initial [Ca] inputs, the amplitude decrease from strong ISM/dry winter to weak ISM/wet winter becomes 21.1% and 25.0%, respectively.

**Figure 5:**
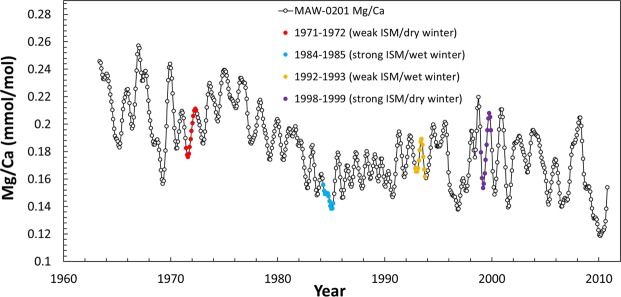
Smoothed MAW-0201 Mg/Ca record with year-long segments used in RMSD amplitude comparisons highlighted. Estimates for the speleothem Mg/Ca values for 1971–1972 (weak ISM/dry winter) are in red, 1984–1985 (strong ISM/wet winter) in blue, 1992–1993 (weak ISM/wet winter) in yellow, and 1998–1999 (strong ISM/dry winter) control in purple. Chronological error is ±1.1–2.7 years. We explore the effect of this error in Table S3.

Variations in amplitude of the measured Mg/Ca in MAW-0201 are generally larger than those predicted by the I-STAL model, suggesting the modeled changes in amplitude are conservative. To explore the relationship between measured rainfall and stalagmite Mg/Ca, we employ the same RMSD amplitude technique to compare three year-long intervals covered by the MAW-0201 record. We use the 1998–1999 strong ISM/dry winter as our control and compare this to the periods of (i) 1971–1972 (weak ISM/dry winter), (ii) 1984–1985 (strong ISM/wet winter), and (iii) 1992–1993 (weak ISM/wet winter). As it is challenging to directly link an individual lamina to a calendar year^39^, we compare year-long segments from the Mg/Ca record to calendar year rainfall bracketed by a ±1 year window that is comparable to the 2*σ* uncertainty of our ^230^Th/U ages (1.1–2.7 years) (Fig. 5). The measured rainfall values for these years and the Mg/Ca amplitudes associated with the ±1 year windows are listed in Table S3. The Mg/Ca amplitudes in MAW-0201 for these example years are highlighted in Fig. 5. During the weak ISM/dry winter period of 1971-1972 the measured amplitude of variation in Mg/Ca is 31% smaller than the control. The Mg/Ca amplitude from the strong ISM/wet winter of 1984–1985 is 73% smaller than the control amplitude. The 1992–1993 weak ISM/wet winter example resulted in a 57% Mg/Ca amplitude decrease relative to the strong ISM/dry winter control (Fig. 5 and Table S3). Even with year-to-year variability and a weak ISM, a larger change in Mg/Ca amplitude is observed during years with increased winter precipitation.

For both modeled and measured Mg/Ca values, the change in the amplitude of the seasonal signal is larger when winter rainfall increases than when ISM rainfall decreases. The forward-modeling results suggest that an increase in winter rainfall of ~375 mm has a larger influence on Mg/Ca amplitude than a decrease in ISM rainfall of >10000 mm (Fig. 4). This relatively low sensitivity to changes in ISM rainfall is most likely related to the very large amount of monsoon rainfall that Meghalaya receives and the resulting low drip interval in Mawmluh Cave, even during years of “weak” ISM rainfall. For example, during 2013, the driest ISM season covered by our record, Sohra still received over 5000 mm of JJAS rainfall. These high rainfall amounts, coupled with higher cave air *p*CO_2_ during the summer, minimizes the influence of PCP on dripwater Mg/Ca and the extent to which this signal is captured in speleothems^40^. Conversely, a proportionally larger rainfall increase during the winter, when cave air *p*CO_2_ is low, will have a significant effect on PCP that would be readily recorded in a speleothem.

To test the sensitivity of Mawmluh Cave speleothem Mg/Ca to in-cave temperature changes, we input median monthly Hanging Gardens temperatures from a 2010–2014 subset of the monitoring study. We exclude 2011–2012, a year of high base temperature in the entire cave potentially caused by reduced temperature buffering capacity of the above soil due to deforestation^23^. This monitoring suggests a temperature variation of up to 2.6 °C in Hanging Gardens. Accounting for this range of temperature variability in the model using the temperature-dependent calcite *D*_Mg_ increases the sensitivity of Mg/Ca amplitude variability to summer rainfall by an additional 0.4% (Tables S4 and S5). This does not change our conclusions due to the small variability in year-to-year monthly Mawmluh Cave temperature. Using the median monthly Mawmluh Cave temperatures increases the sensitivity of Mg/Ca amplitudes to winter rainfall by an additional 0.3%. Using the aragonite *D*_Mg_, we see less sensitivity to temperature variability (Table S5), which is to be expected as the Mawmluh Cave-specific aragonite *D*_Mg_ is not temperature-dependent^33^.

Our trace element results and interpretations are corroborated by the MAW-0201 δ^18^O record. In contrast to the trace element records, the seasonal amplitude of the MAW-0201 δ^18^O record increases during the warm phase of the PDO (Fig. 2). Seasonal differences in MAW-0201 δ^18^O are linked to changes in transport pathways and moisture source^6^. Analysis of winter rainfall at Sohra indicates higher δ^18^O values as a result of shorter and more variable vapor transport pathways than during the ISM^22^.

The increased seasonal amplitude and overall higher δ^18^O values during positive PDO phases have been linked to changes in the ENSO-ISM relationship that lead to more localized moisture transport during the ISM season^6^. The warm phase of PDO and CP El Niño events are correlated with reduced ISM rains in peninsular India^4,8^. However, ISM rainfall in NE India, including the Sohra rainfall record from 1901–2014, does not show a consistent relationship with peninsular Indian rainfall or with PDO and ENSO state^6^. For example, 1974 is the wettest ISM season at Sohra between 1901 and 2014, but is considered a drought year in the All India Summer Monsoon Rainfall record^41^. MAW-0201 δ^18^O does show clear relationships with the PDO, North Pacific Gyre Oscillation, and CP El Niño events, and these relationships have been interpreted to reflect variations in large scale atmospheric circulation, which influence rainfall amounts in peninsular India and moisture transport to NE India^6^. Our new trace element record suggests that the relationship between Mawmluh Cave speleothem isotopic and geochemical records and the PDO may also reflect local winter moisture conditions.

The sensitivity of Mawmluh Cave trace element proxies to dry season conditions offers the opportunity to investigate the influence of ocean-atmosphere oscillations such as ENSO and PDO on winter rainfall. Deeper understanding of these linkages can improve both our reconstructions of past climates and our ability to anticipate future rainfall variability. An analysis of monthly winter rainfall between 1925 and 1998 revealed a modest negative relationship between PDO, ENSO, and rainfall across peninsular India due to a shift of the Hadley cell’s descending arm over central India^11^. The same study showed that NE Indian rainfall is most closely, and positively, linked to sea surface temperature (SST) in the Bay of Bengal, but found a weak negative relationship with PDO phase. The positive relationship between winter rainfall and Bay of Bengal SST anomalies is explained by the formation of a Rossby-type atmospheric wave with a low-pressure trough over NE India. The low-pressure trough allows convection and increased rainfall in NE India when Bay of Bengal SST is higher. This analysis used a low spatial resolution rainfall dataset based on an 18-cell 5° grid covering the Indian subcontinent^42^. Northeastern India, however, is characterized by dramatic variability in elevation and climate that is likely not captured by a coarse grid^43^. In contrast, our analysis, based on observations at a meteorological station suggests a measurable increase in winter rainfall at Sohra during the positive phase of the PDO (Fig. S4). Our results are consistent with the observation that El Niño events are associated with an intensified subtropical westerly jet, also due to the intensification of the descending arm of the Hadley cell over central India^12^, which would strengthen western disturbances that deliver winter rainfall to northern India^12,44^. Higher winter rainfall due to strengthened western disturbances would induce lower Mg/Ca and higher δ^18^O values in speleothems deposited during the corresponding winter months.

Seasonal bias in speleothem growth due to ventilation is widely observed^27,45^. Although monsoonal regions are primarily located in the tropics and subtropics where seasonal temperature fluctuations are relatively small, temperature driven ventilation can still lead to seasonal speleothem growth rate changes^23,35,46^. Seasonal changes in cave ventilation and speleothem growth can also arise via other mechanisms, such as changes in prevailing winds, which must be considered when interpreting proxy records^47^. Our record suggests that speleothems from monsoonal regions can record growth and proxy information that is biased toward the dry season, even with rainfall volumes orders of magnitude lower than wet season rainfall. The role of dry season rainfall in driving proxy variability may be especially important to consider when interpreting records from slower growing speleothems where carbonate cannot be sampled at seasonal resolution.

## Conclusions

Our results suggest that the amplitude of seasonal variations in speleothem trace element to calcium ratios reflect the seasonality of rainfall. Dry season rainfall is likely an important driver of variability in the seasonal amplitudes of PCP-sensitive proxies such as Mg/Ca and U/Ca, but also δ^18^O, which is sensitive to seasonal differences in moisture transport. This is especially true in seasonally ventilated caves where speleothem growth is biased towards the dry season. Thus, our new record supports a more cautious approach toward the interpretation of speleothem isotopic and geochemical records as primarily summer monsoon intensity or annual rainfall indicators. In the hydrologically extreme region of NE India, even a weak ISM delivers 1000s of mm of rainfall. The PCP process that drives variability in speleothem trace elements may not be sufficiently sensitive to rainfall variability around such high mean values, especially when wet season cave air *p*CO_2_ is high. Analysis of dripwater and modern speleothems from regions where the seasonal difference in rainfall or the amount of monsoon season rain is lower, or where the ventilation regime is different, would provide an opportunity to test the applicability of these results to other settings.

Winter rainfall in NE India and corresponding speleothem isotopic and geochemical records are likely affected by Pacific decadal variability. Increasing the number and longevity of seasonally-resolved speleothem records will allow us to further investigate these precipitation-proxy relationships in the past. Future isotope-enabled modeling may help to determine the relative influence of ISM moisture transport and winter rainfall variability on speleothem δ^18^O. Understanding past dry season rainfall variability may help improve mediation strategies against drought before it impacts the rainiest place on earth.

## Methods

### ^230^Th-U dating

Detailed methods for ^230^Th-U dating and δ^18^O analysis of MAW-0201 were previously described in Myers *et al*.^6^. The age model chosen uses six U-series dates and 1000 Monte Carlo simulations with polynomial interpolation in the MATLAB COPRA package^48^. Although the MAW-0201 laminae appear to be annual and the U-series dates correspond well with the layer count ages, layer counting was not utilized in COPRA given the possibility of skipped years or multiple laminae being deposited in one year^39^ and the observation that COPRA may underestimate uncertainty on the age model when layer counts are included.

### Trace element analysis

For this study, trace element concentrations were measured along the growth axis of a thin section of MAW-0201 using laser ablation with a Photon Machines Excimer 193 nm laser coupled to a ThermoFisher iCAP Qc quadrupole ICP-MS at Vanderbilt University. Laser ablation was performed using a 20 μm × 100 μm rectangular aperture at 20% laser power using a repetition rate of 15 Hz and a scan speed of 5 μm/s. The line scans followed a pre-ablation step conducted over the sample path at a speed of 10 μm/s at 50% laser power and a repetition rate of 15 Hz. The multi-element synthetic glass standard, NIST SRM 612, and the MACS3 synthetic pressed aragonite powder were analyzed at the beginning and end of the run, and NIST SRM 612 was used for elemental quantification. Raw data were processed using the Iolite software package and then converted to ratios relative to calcium (mmol/mol)^49,50^.

In order to compare the MAW-0201 trace element ratios with instrumental climate data, a Gaussian kernel with bandwidth 0.15 was used to smooth and down-sample the laser ablation data to a monthly time step without oversmoothing and losing seasonal variability (Fig. S9)^51^. In analyzing the Mg data, points higher than 2 standard deviations from the mean were removed prior to smoothing (98 values above 80.3 ppm Mg). These high Mg measurements appeared as spikes in the time-series and were likely derived from micron-scale soil or bedrock particles embedded in the stalagmite^52,53^. With the smoothed time series for each trace element ratio, we performed continuous wavelet transforms (CWT) which visualize the different periodicities that form the overall record in a frequency-time space using the wavelet coherence toolbox for MATLAB^54^. We used the Morlet wavelet for all analyses and pad the ends of each time series with the mean value of the data to reduce edge effects from the cone of influence.

### I-STAL model setup

The I-STAL speleothem forward model was used to investigate potential mechanisms for observed trace element variations in MAW-0201^38^. The Mawmluh Cave monitoring study from 2007–2014 provides cave *p*CO_2_, air temperature, and drip rate data to utilize in the model. However, monitoring data are limited during the ISM season as flooding in some passages prevents access to the cave^23^. Therefore, two measurements of cave air *p*CO_2_ taken in May 2010 and May 2012 (534 and 1049 ppm) were used to estimate summer cave air *p*CO_2_. While there is a large difference in these two May measurements, we choose to use the 1049 ppm value as it was taken in Hanging Gardens, the room where MAW-0201 grew. We estimated monthly *p*CO_2_ for the rest of the year from available data during January and February, averaging 467 ppm CO_2_ (n = 11). All *p*CO_2_ inputs are listed in Table S4. The temperature in the Hanging Gardens passage of Mawmluh Cave over a subset of the monitoring period (2010–2014) ranged from 16 °C to 23 °C while temperature at the surface varied between <1 °C and 30 °C. Sensitivity tests using observed seasonal temperature variability in Mawmluh Cave indicate that temperature does not have a significant influence on trace element variability in the I-STAL model (Fig. S10) relative to the influence of rainfall (drip interval) variability. Thus, temperature is held constant at 19 °C, the average temperature measured in the Hanging Gardens passage between 2010 and 2014^23^. Initial [Ca] will vary based on the degree of dissolution in the epikarst and is thus variable with time and surface conditions such as soil *p*CO_2_ ^38^. Using [Ca] simplifies the I-STAL inputs, integrating parameters that affect hostrock dissolution like soil *p*CO_2_, flow path length, and mixing of waters into one. We use the median value of all measured Mawmluh Cave dripwater Ca concentrations (153 ppm) (Fig. S5) as the initial [Ca] input to reflect a modest amount of water-rock interaction and dissolution, allowing us to focus primarily on the effects of PCP on Mg/Ca. To explore the sensitivity of the model to initial dripwater [Ca], we also test the minimum and maximum (111 and 420 ppm) measured values of Mawmluh Cave dripwater [Ca].

## Supplementary information

Supplementary Information
