# Supplementary material for: Sensitivity of speleothem records in the Indian Summer Monsoon region to dry season infiltration
Source: Sci Rep. 2019 Mar 25;9:5091. doi: 10.1038/s41598-019-41630-2 (PMC6434041; doi:10.1038/s41598-019-41630-2)
Supplement: Supplementary file 1 — Supplementary Information [file 41598_2019_41630_MOESM1_ESM.pdf]

Supplementary information for

Sensitivity of speleothem records in the Indian Summer Monsoon region to dry season infiltration

Elli R. Ronay[1], Sebastian F.M. Breitenbach[2], Jessica L. Oster[1]

[1]Department of Earth and Environmental Sciences, Vanderbilt University, Nashville, TN

[2]Institute for Geology, Mineralogy & Geophysics, Ruhr-University Bochum, Bochum, Germany

Corresponding author: Elli Ronay (elli.ronay@vanderbilt.edu)

Contents of this file

Figure S1: Map of Mawmluh Cave
Figure S2: Sr/Ca and Ba/Ca records
Figure S3: Annual Sohra rainfall regression
Figure S4: December precipitation moving average and z-score
Figure S5: Dripwater ^{44}Ca data
Figure S6: Drip interval vs. rainfall
Figure S7: Monthly Sohra rainfall and I-STAL input rainfalls
Figure S8: RMSD process
Figure S9: Trace element Gaussian kernel smoothing example
Figure S10: Temperature sensitivity experiments
Figure S11: CWTs of JJAS and NDJF Sohra rainfall

Table S1: Correlation coefficients for trace element ratios to calcium
Table S2: I-STAL input parameters
Table S3: MAW-0201 Mg/Ca amplitude analysis and real-world rainfall data
Table S4: I-STAL results with D_{Mg} for aragonite and calcite
Table S5: Temperature sensitivity I-STAL results with D_{Mg} for aragonite and calcite

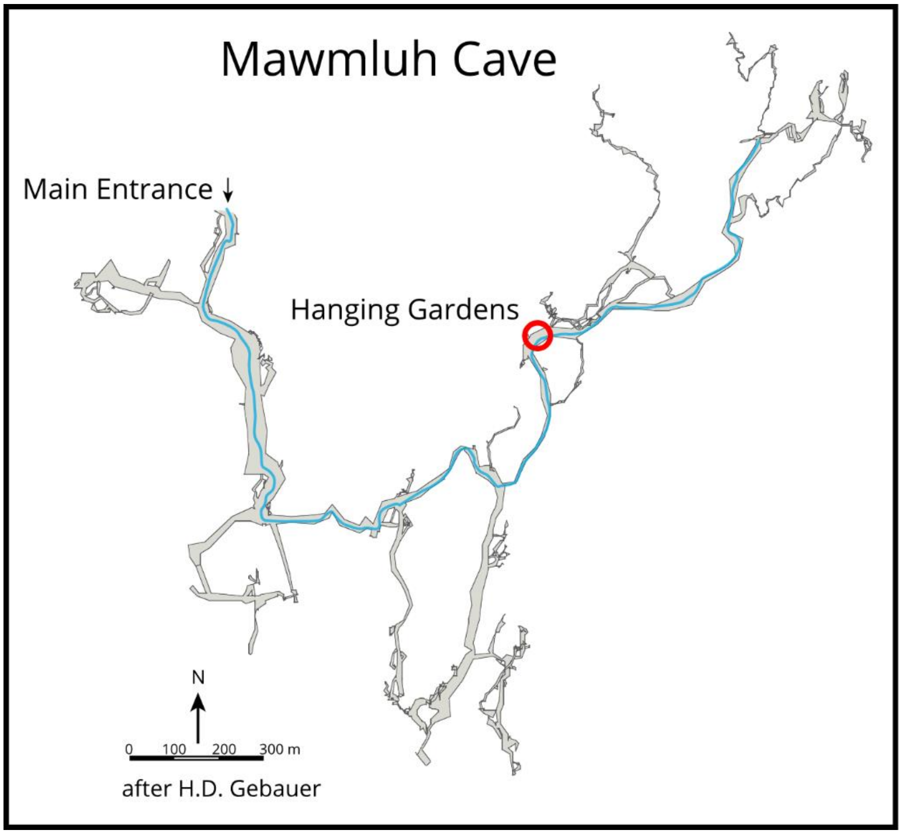

Figure S1. Map of Mawmluh Cave adapted from Breitenbach et al. (2015). Speleothem MAW-0201 was sampled from the Hanging Gardens passage. The blue line represents the river flowing through Mawmluh Cave.

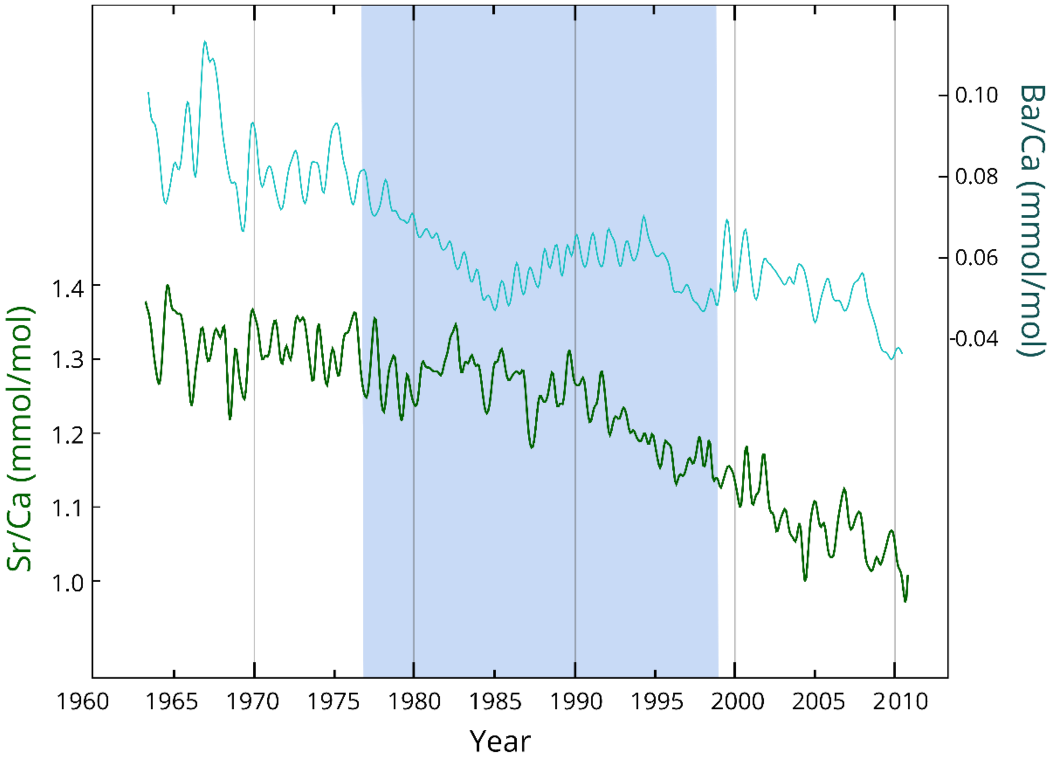

Figure S2. Smoothed MAW-0201 Sr/Ca and Ba/Ca (mmol/mol) time series. Blue shading indicates positive PDO period.

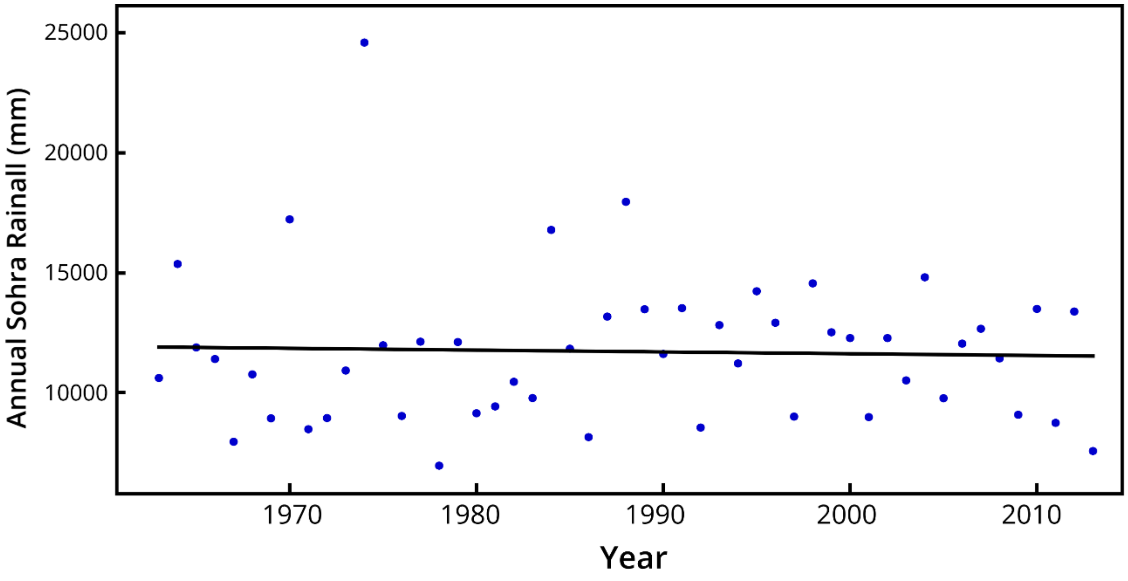

Figure S3. Annual Sohra rainfall data over the period covered by the MAW-0201 record plotted with linear regression. No significant trend is present. The regression equation is y = -7.4896x + 26589;
R^2 = 0.0013.

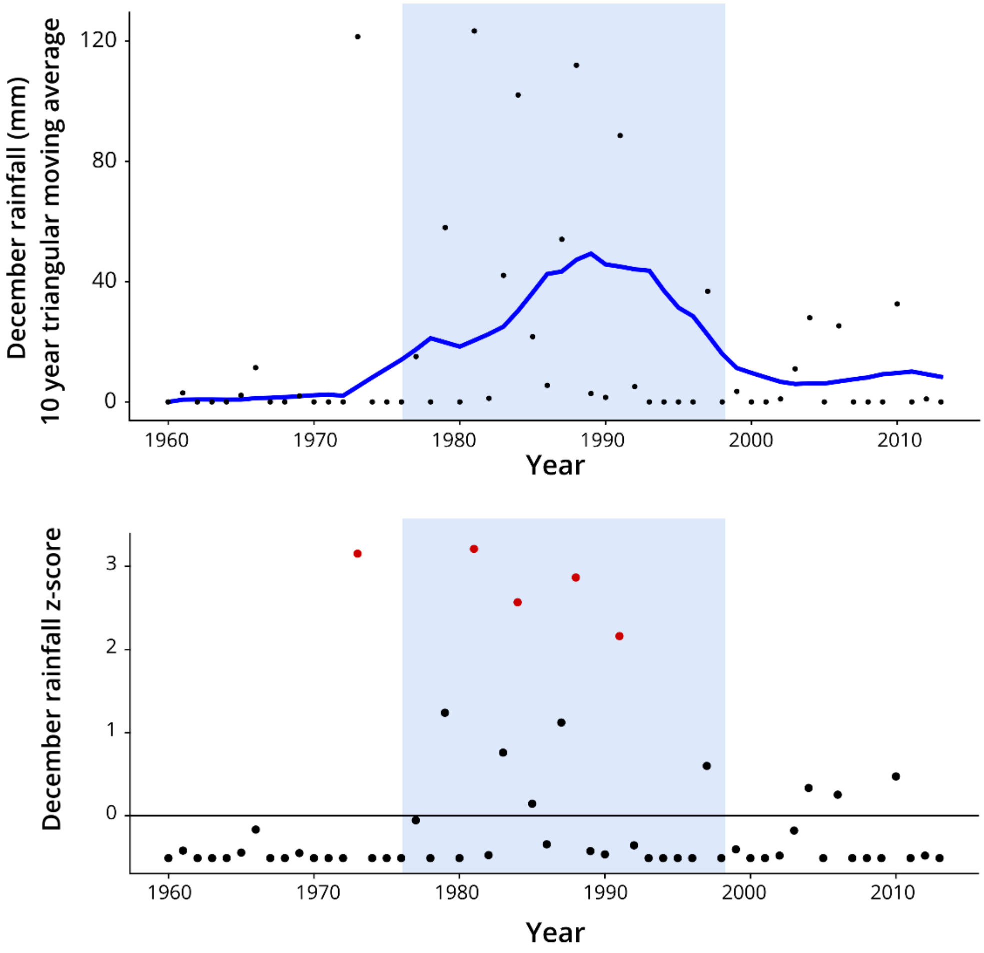

Figure S4. Triangular moving average and z-score of December precipitation (mm) throughout the MAW-0201 record. Red points are above 2 standard deviations from mean December rainfall. Blue highlight indicates positive PDO period.

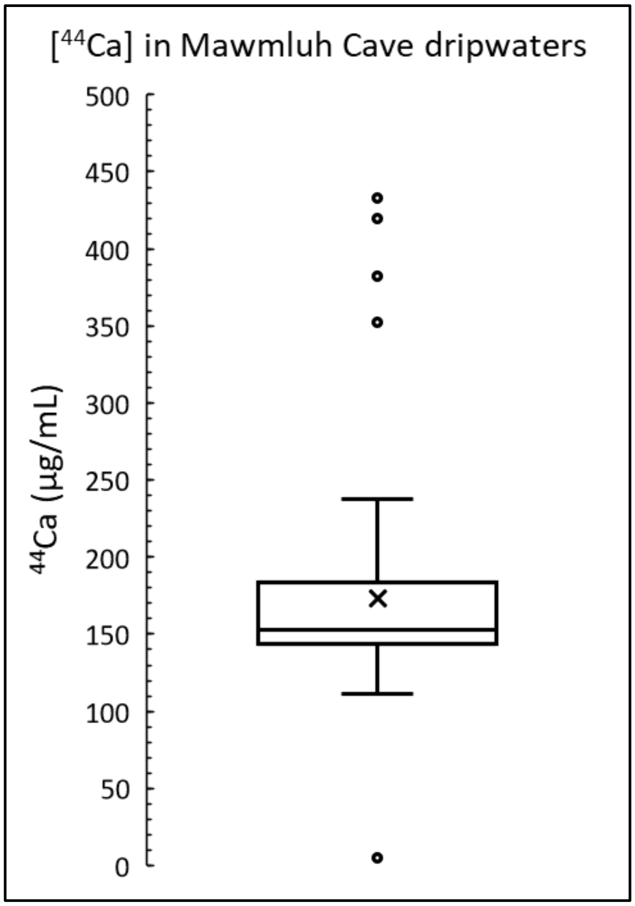

Figure S5. Box and whisker plot of 59 ICP-MS ^{44}Ca measurements for Mawmluh Cave dripwater collected between 2011 and 2014. Excluding one 5 µg/mL (ppm) outlier, the minimum ^{44}Ca concentration is 111, median is 153, and maximum is 420 µg/mL.

These dripwater data were originally collected for stable isotope measurements, not for cation analysis, and so collection methods varied from accepted trace element collection protocol. Specifically, the samples were not acidified in the field and sat unacidified for 3-6 years. Therefore, these cation measurements allow us to estimate initial Ca concentrations, but we do not interpret them further.

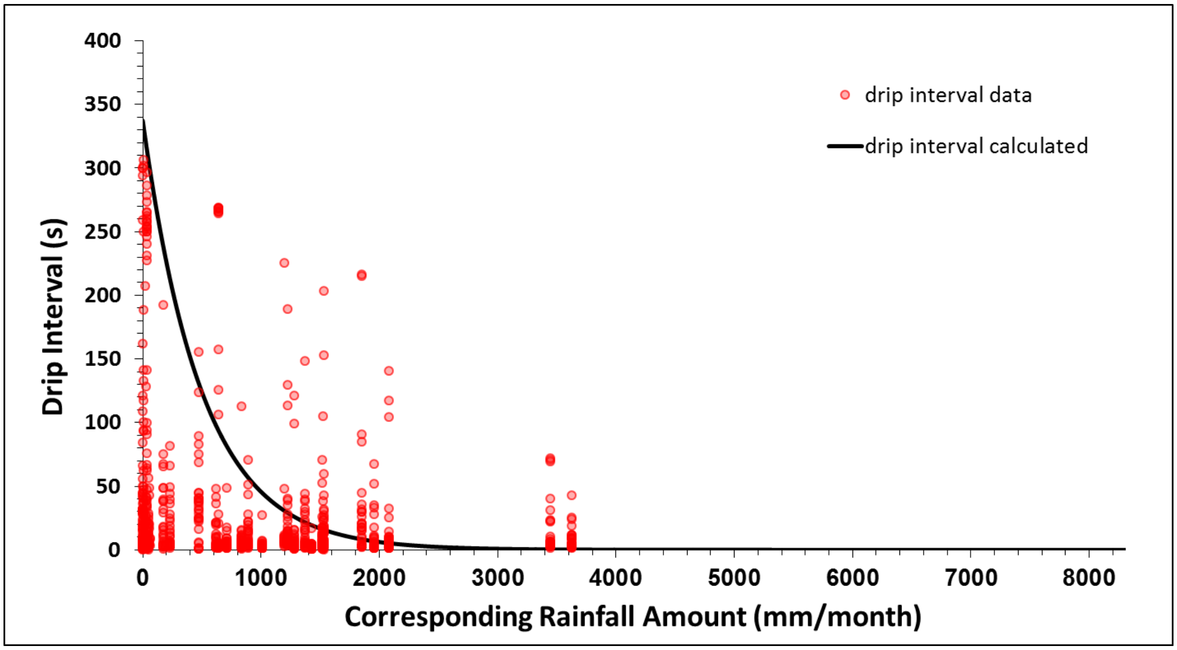

Figure S6. Drip interval for I-STAL input calculated from drip rate (Breitenbach et al., 2015) and rainfall data (http://www.cherrapunjee.com/weather-info/). Red dots reflect all drip intervals from Breitenbach et al. (2015) Candle Highway drip B, recorded during months with total rainfall falling on the x axis.

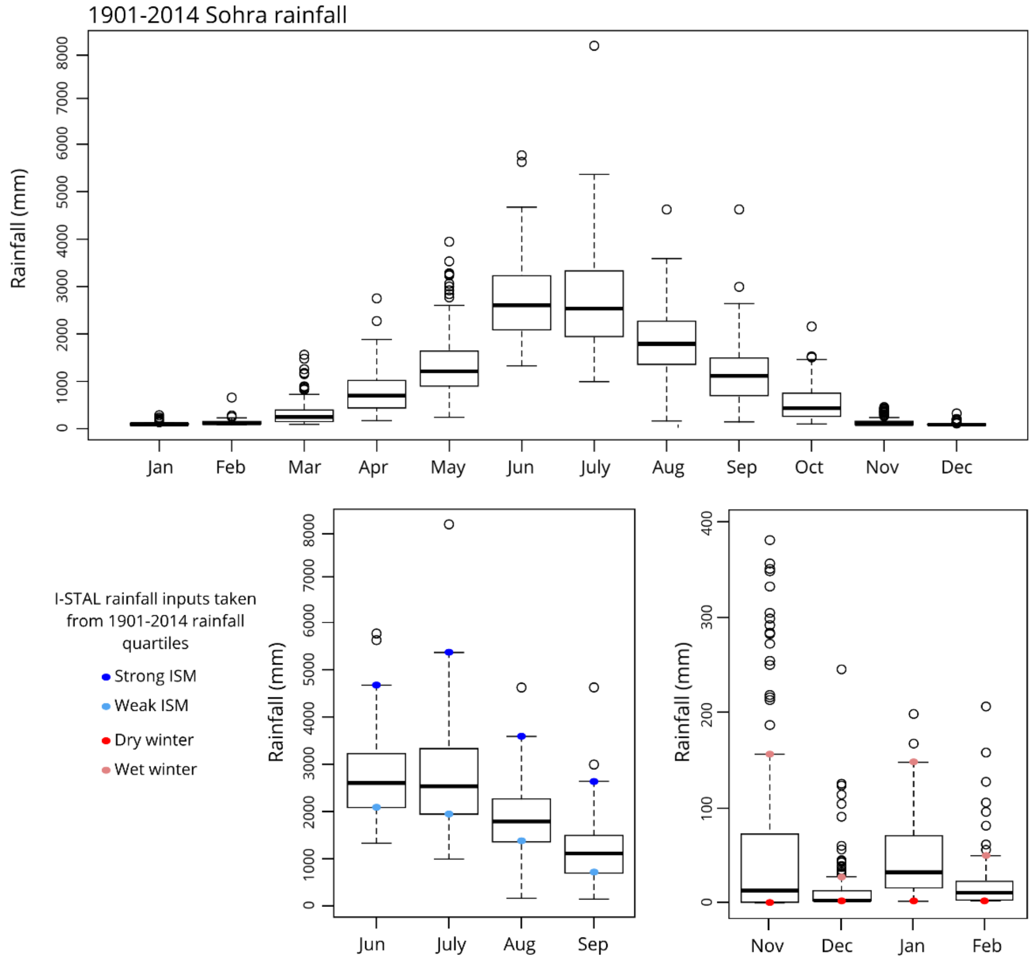

Figure S7. Top: Box-and-whisker plot of monthly Sohra rainfall from 1901-2014. Bottom: Rainfall amounts corresponding to I-STAL drip interval inputs for strong and weak ISMs (left) and wet and dry winters (right) plotted on top of Sohra rainfall to show differences between modeled scenarios.

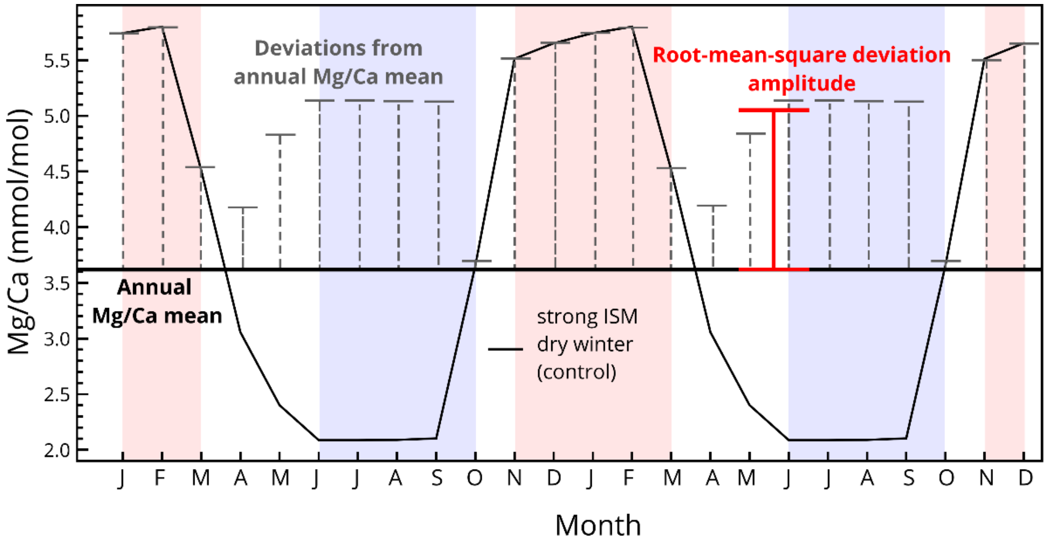

Figure S8. Amplitude calculation using the root-mean-square deviation (RMSD) from the annual mean (horizontal black line). Monthly deviations from the mean (grey dotted lines) are squared, then averaged. We use the root of this mean of square deviations (RMSD, red line) as the Mg/Ca amplitude.

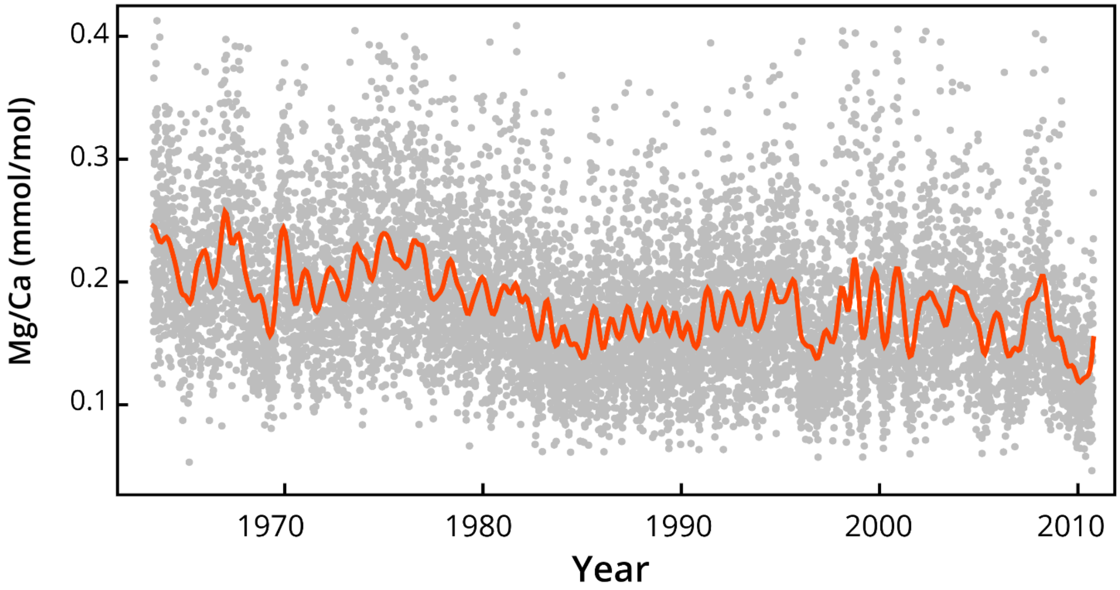

Figure S9. Smoothed LA ICP-MS time series (red line) using a Gaussian kernel smoother with bandwidth 0.15. Grey points indicate full dataset.

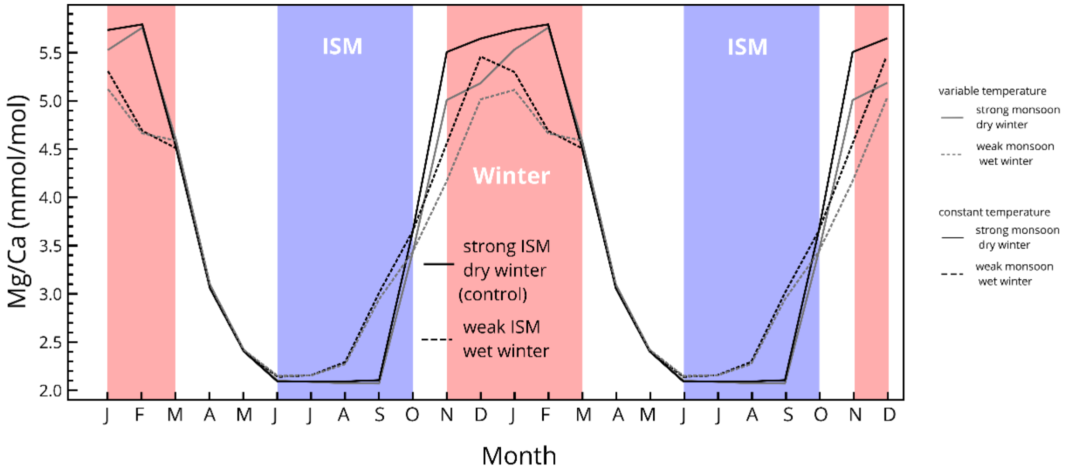

Figure S10. Temperature sensitivity experiment using D_{Mg} for aragonite (Wassenburg et al., 2016). Grey lines are experiments in which I-STAL temperature input is varied throughout the year using Breitenbach et al. (2015) observational data.

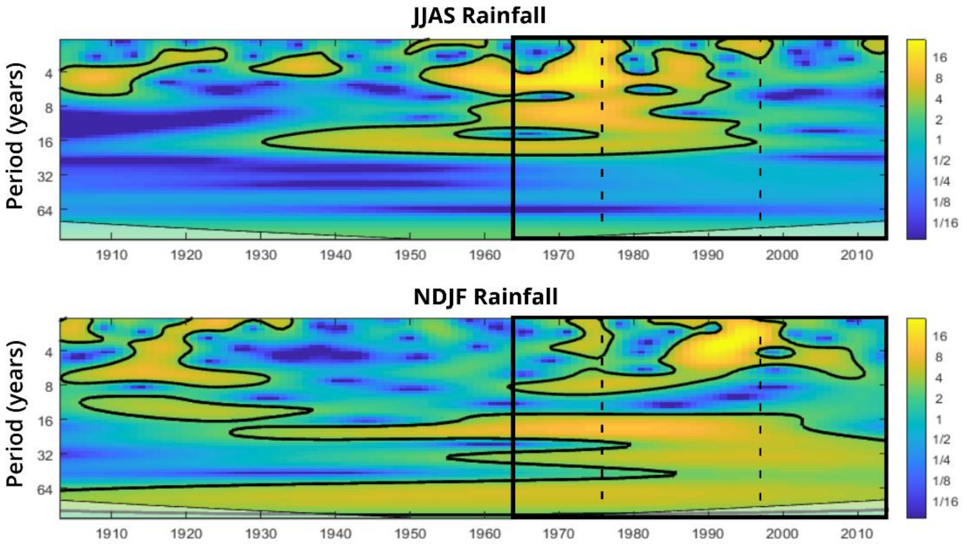

Figure S11. Continuous wavelet transforms (CWTs) of ISM (top) and winter (bottom) rainfall from Sohra for 1901 to 2014. Our record falls within the black box and the positive Pacific Decadal Oscillation (PDO) interval is within the dotted lines. More yellow colors reflect stronger periodicities, periodicities significant at the 95% confidence interval are outlined in black. Periodicities > 16 years, potentially related to PDO, are seen in the NDJF rainfall, but not in ISM rainfall.

Table S1. Correlation coefficients for trace element ratios to calcium in stalagmite MAW-0201. Values significant at the 95% confidence interval are marked with an asterisk (*). All element ratios are positively correlated and significant, except Sr/Ca and U/Ca.

	Mg/Ca	Ba/Ca	Sr/Ca
Ba/Ca	0.85*		
Sr/Ca	0.49*	0.70*	
U/Ca	0.54*	0.48*	0.035

Table S2. Input parameters (drip interval, rainfall and pCO_2) for the I-STAL modeling experiment.

	drip interval (s)	rainfall (mm)	pCO_2 (ppm)
Strong ISM	0.007-1.8	2622-5420	1050-1060
Weak ISM	5.6-96	629-2053	1050-1060
Dry winter	337-350	0	400-500
Wet winter	247-321	25-55	400-500

Table S3. Summary of MAW-0201 Mg/Ca real-world seasonal amplitude investigation. SMDW: strong Indian Summer Monsoon (ISM) and dry winter.

	Year	ISM rainfall (mm)	Winter rainfall (mm)	Amplitude difference from SMDW	- 1 year	+ 1 year	Average difference
Strong ISM Dry Winter	1998-1999	11632	0	0%	-31%	+23%	-3%
Strong ISM Wet Winter	1984-1985	11372	148	-73%	-33%	-41%	-49%
Weak ISM Wet Winter	1992-1993	6210	717	-57%	-43%	-23%	-41%
Weak ISM Dry Winter	1971-1972	6915	0.2	-31%	-49%	-10%	-30%

Table S4. Results from the I-STAL modeling experiments using D_{Mg} for aragonite (Wassenburg et al., 2016) and calcite (Day & Henderson, 2013). Blue highlights ISM months and red highlights winter months. SMDW: strong ISM and dry winter, SMWW: strong ISM and wet winter, WMDW: weak ISM and dry winter, WMWW: weak ISM and wet winter.

I-STAL Results using [Ca] = 153 ppm

Month	pCO_2	Temperature	$SMDW_{Calcite}$ Mg/Ca	$SMDW_{Aragonite}$ Mg/Ca	$WMDW_{Calcite}$ Mg/Ca	$WMDW_{Aragonite}$ Mg/Ca	$SMWW_{Calcite}$ Mg/Ca	$SMWW_{Aragonite}$ Mg/Ca	$WMWW_{Calcite}$ Mg/Ca	$WMWW_{Aragonite}$ Mg/Ca
1	400	19	5.7425	0.0285	5.7425	0.0285	5.3047	0.0263	5.3047	0.0263
2	400	19	5.7994	0.0287	5.7994	0.0287	4.6932	0.0232	4.6932	0.0232
3	500	19	4.5124	0.0223	4.5124	0.0223	4.5124	0.0223	4.5124	0.0223
4	700	19	3.0568	0.0150	3.0568	0.0150	3.0568	0.0150	3.0568	0.0150
5	1000	19	2.4013	0.0118	2.4013	0.0118	2.4013	0.0118	2.4013	0.0118
6	1050	19	2.0872	0.0102	2.1370	0.0104	2.0872	0.0102	2.1370	0.0104
7	1060	19	2.0870	0.0102	2.1540	0.0105	2.0870	0.0102	2.1540	0.0105
8	1050	19	2.0892	0.0102	2.2919	0.0112	2.0892	0.0102	2.2919	0.0112
9	1000	19	2.1029	0.0103	3.0113	0.0148	2.1029	0.0103	3.0113	0.0148
10	700	19	3.6557	0.0180	3.6557	0.0180	3.6557	0.0180	3.6557	0.0180
11	500	19	5.5121	0.0273	5.5121	0.0273	4.5633	0.0226	4.5633	0.0226
12	400	19	5.6514	0.0280	5.6514	0.0280	5.4651	0.0271	5.4651	0.0271
Percent decrease from SMDW			0%	0%	5.8%	5.8%	16.5%	16.5%	22.3%	22.3%

Table S5. Results from the I-STAL temperature sensitivity modeling experiments using D_{Mg} for aragonite (Wassenburg et al., 2016) and calcite (Day & Henderson, 2013). Blue highlights ISM months and red highlights winter months. Abbreviations as in Table S4.

Temperature sensitivity results using [Ca] = 153 ppm

Month	pCO_2	Temperature	$SMDW_{Calcite}$ Mg/Ca	$SMDW_{Aragonite}$ Mg/Ca	$WMDW_{Calcite}$ Mg/Ca	$WMDW_{Aragonite}$ Mg/Ca	$SMWW_{Calcite}$ Mg/Ca	$SMWW_{Aragonite}$ Mg/Ca	$WMWW_{Calcite}$ Mg/Ca	$WMWW_{Aragonite}$ Mg/Ca
1	400	18.0	5.5377	0.0280	5.5377	0.0280	5.1187	0.0259	5.1187	0.0259
2	400	18.8	5.7656	0.0287	5.7656	0.0287	4.6673	0.0231	4.6673	0.0231
3	500	19.5	4.5915	0.0225	4.5915	0.0225	4.5915	0.0225	4.5915	0.0225
4	700	19.5	3.0952	0.0151	3.0952	0.0151	3.0952	0.0151	3.0952	0.0151
5	1000	19.3	2.4177	0.0118	2.4177	0.0118	2.4177	0.0118	2.4177	0.0118
6	1050	19.2	2.0964	0.0102	2.1467	0.0104	2.0964	0.0102	2.1467	0.0104
7	1060	18.9	2.0848	0.0102	2.1516	0.0105	2.0848	0.0102	2.1516	0.0105
8	1050	18.6	2.0708	0.0102	2.2699	0.0112	2.0708	0.0102	2.2699	0.0112
9	1000	18.2	2.0678	0.0103	2.9443	0.0147	2.0678	0.0103	2.9443	0.0147
10	700	17.0	3.4386	0.0176	3.4386	0.0176	3.4386	0.0176	3.4386	0.0176
11	500	16.3	5.0128	0.0262	5.0128	0.0262	4.1717	0.0217	4.1717	0.0217
12	400	16.6	5.1879	0.0269	5.1879	0.0269	5.0202	0.0261	5.0202	0.0261
Percent decrease from SMDW			0%	0%	6.2%	5.9%	16.8%	16.6%	23.0%	22.5%
